# O006. Efficacy of prophylactic therapy in chronic primary headache with use of biofeedback

**DOI:** 10.1186/1129-2377-16-S1-A134

**Published:** 2015-09-28

**Authors:** Biagio Ciccone, Luigi Balzano, Giacinta D'Otolo

**Affiliations:** Ambulatorio ATHENA, Saviano, NA Italy; ASL NA3 SUD, Naples, Italy; Ambulatorio Athena, Saviano, NA Italy

## Introduction

Retrospective study of patients with chronic tension headache (CTH) and chronic migraine (CM).

## Objective

To compare the efficacy of biofeedback (BFB) compared to only prophylactic therapy in these primary headaches [1–4].

## Materials and methods

We evaluated a total of 8 patients with CTH and 8 patients with CM. All patients had a history of primary headache and had never undergone prophylactic therapy. The observation period lasted 90 days. Four CTH patients and 4 CM patients underwent only prophylactic therapy (amitriptyline 20 mg daily), the remaining 4 CTH and 4 CM prophylactic therapy and BFB training sessions. Assessment tools outcome measures were:

- Headache diary to assess days per month with headache;
- Analgesic consumption and/or triptans;
- Score of the visual analogue pain scale (VAS);
- SEMG parameter for patients who carried out BFB training.

## Results

At the end of the 90 day observational period there was a significant improvement (reduction in headache days per month, in VAS score, in analgesic consumption and in SEMG parameter) in CTH and CM patients that had undergone both BFB training and prophylactic therapy when compared to the group of patients treated only with prophylactic therapy drug.

## Discussion and conclusions

The overall data confirmed the efficacy of the BFB training in the prophylaxis of primary headaches, further supporting the benefits already possible with the therapy of only pharmacological prophylaxis (Table 1). The data also showed a clear dominance of efficacy, especially in the forms of chronic tension headache (Table 2).

**Table 1:** Overall differences between the two groups after 90 days of therapy.

	Frequency	VAS	Analgesic consumption	Triptan consumption	SEMG
CTH	-58%	-37%	-62%		
**CTH BFB**	**-75%**	**-67%**	**-86%**		**-54%**
**CM**	**-53%**	**-34%**	**-60%**	**-50%**	
**CM BFB**	**-61%**	**-43%**	**-75%**	**-63%**	**-54%**

**Table 2:** Differences between CTH and CM in treatment with BFB after 90 days of therapy.

	Frequency	VAS	Analgesic consumption	SEMG
CTH BFB	-75%	-67%	-86%	-54%
**CM BFB**	**-61%**	**-43%**	**-75%**	**-54%**
**Difference CTH BFB and CM BFB**	**-14%**	**-24%**	**-11%**	**-50%**

Written informed consent to publication was obtained from the patient(s).
